# Possible Measures to Improve Both Participation and Response Quality in Japan’s National Health and Nutrition Survey: Results from a Workshop by Local Government Personnel in Charge of the Survey

**DOI:** 10.3390/nu14193906

**Published:** 2022-09-21

**Authors:** Midori Ishikawa, Tetsuji Yokoyama, Hidemi Takimoto

**Affiliations:** 1 Department of Health Promotion, National Institute of Public Health, Saitama 351-0197, Japan; 2 Department of Nutritional Epidemiology and Shokuiku, National Institutes of Biomedical Innovation, Health and Nutrition, Tokyo 162-8636, Japan

**Keywords:** National Health and Nutrition Survey, participation rate, local government health personnel, workshop, category

## Abstract

Increasing participation rates are crucial to ensure the representativeness of national survey results of the population. This study aimed to identify measures that could be taken by local government personnel in charge of the National Health and Nutrition Survey (NHNS), Japan, to improve participation rates. The subjects were twenty-one health personnel who worked in 19 local governments and participated in the training course at the National Institute of Public Health. Qualitative data were collected through a workshop. They discussed the problems that seem to affect participation rates and identified possible solutions. The contents were coded and grouped to create categories, using the Jiro Kawakita (KJ) method. For data analysis, researchers combined and reviewed all codes and categories. The measures that could improve participation rates were divided into the following 12 categories: 1. standardization of survey methods, 2. investigator skills, 3. survey organization, 4. venue setting, 5. accessing target households, 6. time of survey, 7. responses during the investigation, 8. confirming meal contents reported in the nutritional intake status survey, 9. rewards/incentives, 10. possible rewards, 11. feedback on survey results, and 12. survey practices during the COVID-19 pandemic. These findings represent viable initiatives for local health personnel to increase participation rates for the NHNS.

## 1. Introduction

The majority of health and nutritional data of the population are collected through health surveys [1]. However, the representativeness of this data is threatened by the persistent decline in participation rates among the selected subjects in these surveys [2,3,4]. Low participation rates may have serious implications for the generalizability of survey results and the accuracy of any conclusions based on survey data. We must identify methods to increase participation rates while also improving the distribution of responses to ensure they are representative of the population [5,6].

Declining participation rates have become a major concern in national surveys across several countries in recent years [7,8]. One of the reasons for this is the increasing number of surveys and research studies conducted in the general population [9]. The frequent invitations to participate in different studies may increase the likelihood that eligible participants may ultimately decline to do so due to research fatigue [10]. Other reasons include the rise in telemarketing, concerns about privacy and confidentiality, decreased interest in social contribution, and a general decline in volunteerism [11,12]. For longitudinal or cohort studies that reevaluate a single sample over time, survey participation might decrease with each follow-up because of waning interest in the subject matter of the study [13,14].

One example of a survey that has been struggling with falling participation rates is the National Health and Nutrition Survey (NHNS), which is implemented annually in Japan. The NHNS was first introduced in 1945 after World War II to assess the nutritional status and dietary habits of Japanese residents [15], which had then been conducted by Japan’s Ministry of Health, Labour and Welfare (MHLW). The high participation rates in this survey ensured the reliability of its findings, which are subsequently used for planning and evaluating national policies related to health. However, the participation rates in this survey have been declining in recent years. The rate was 70.2% in 2003 and 66.5% in 2007 [16]. This is especially true for younger adults, which has affected the overall survey results [17]. Additionally, lifestyle characteristics in Japan have been changing in recent years, with reports showing an increase in the number of people living alone and living in apartments instead of houses [18,19]. Surveys must be constantly revised to adapt to these and other lifestyle changes, and while the MHLW continually updates the research protocol and manual [20], the participation rate is still gradually declining. This limits the amount of information obtained with every new edition or update of the survey.

The NHNS is conducted by public health centers installed in 47 prefectures and 110 cities and special wards (Supplementary Figure S1) [15]. In each public health center, the personnel responsible for conducting the survey are constantly challenged to think of alternatives to improve the participation rate of the survey, depending on the situation of each local resident. The NHNS survey includes (1) a Nutritional Intake Status survey conducted through home visits, (2) a Physical Status Questionnaire that must be conducted in a specific venue (for physical measurement of height, body weight, abdominal circumference, blood pressure, blood tests, and a medical interview), and (3) a Lifestyle Habits Questionnaire conducted through home visits or online. Investigators and the personnel in charge of the survey need to find measures they can take to improve the on-site participation rate.

By combining and analyzing the efforts of survey teams across different regions, new methods may be found that can be shared, to improve the participation rate of the NHNS. Increasing both participation rates and quality of responses might be important. However, few reports have used this methodology to evaluate the actions that could be taken to improve participation rates in the survey.

In other countries, several studies have suggested measures for improving survey participation and response rates. Studies have investigated, for instance, whether more responses were received via mail or online surveys and what kind of incentives could be effective in rewarding survey participation [21,22,23]. The proposals for measures to improve the survey participation rate in Japan’s NHNS could be beneficial to other countries and to ensure the long-term reliability of the results of NHNS.

Every year, the National Institute of Public Health (NIPH) in Japan conducts a training course for health personnel of the local government to improve their knowledge and skills related to the NHNS [24]. The course included a workshop in fiscal years 2018–2020, wherein health personnel received a lecture on survey participation rates and worked in groups to consider possible means of improving participation rates. They discussed and categorized the problems that seemed to affect survey participation rates and identified the possible solutions from their prior experiences. A qualitative analysis of the data obtained from this workshop could provide high-quality insights with practical applications for improving participation rates [25]. This study was conducted based on the data collected in workshops held in fiscal year 2020.

## 2. Materials and Methods

### 2.1. Context of Study

Before conducting the present study, we were responsible for three annual workshops that we used as a basis to develop our research materials. The study design and workshop contents were developed based on those investigations and materials [26].

### 2.2. Study Subjects and Procedure

The subjects were the health personnel including registered dietitians and public health nurses who worked in 19 local governments and participated in the training course at the NIPH. The training objectives and curriculum were presented on the NIPH website, and the training course pamphlet was mailed to all local governments (47 prefectures, and 110 cities and special wards).

The training was held for 4 days in February 2021, and the workshop was included on the second day of the curriculum [26]. On this date, participants would attend a lecture on survey participation rates and work in groups to discuss possible means of improving participation rates.

Twenty-two local governments expressed interest in and registered to attend the training. Next, we sent a request letter, including the purpose of this study and a semistructured questionnaire, to the headquarters of each of these 22 governments.

In the semistructured questionnaire, we asked the institutions and public health centers in charge of the survey the following questions: “What problems seem to be affecting the participation rate of the National Health and Nutrition Survey?” and “What are possible measures to improve the participation rate of the survey?” Additionally, the health personnel were asked about their qualifications (e.g., dietitian, public health nurse) and years of administrative experience. Twenty-one personnel from 19 local governments agreed to cooperate with this study, and written informed consent was obtained from all health personnel.

The study involved the following steps: 1. each workshop member provided their opinion on the problems and solutions regarding falling survey participation rates; 2. members shared and discussed their opinions with members of other local governments; 3. members combined their opinions and insights into a matrix; 4. members analyzed the matrices and coded the information based on its contents; 5. workshop members presented their results.

The workshop ran for 140 min and was in Japanese. Further details on its procedures and contents are shown in Table 1.

Firstly, we implemented the workshop using online platform (Zoom Video Communications, Inc., San Jose, CA, USA) because it was difficult to conduct face-to-face meetings while COVID-19 restrictions were in place. The first part of the workshop involved skill acquisition, such as speaking with microphone and screen sharing, and group work exercises. Then, based on the contents of the semistructured questionnaire that the health personnel answered before the training, the following topics were discussed: (1) problems that seem to affect the participation rate of the NHNS and (2) measures that could be used to improve the participation rate. Each member filled out a worksheet with their opinions on these issues. They were asked to write clearly and unambiguously, and to be as specific as possible (for example, writing “health department manager” instead of “boss”). Next, they carried out an online group exercise based on the two abovementioned topics. Five groups of 4–5 persons were created based on each individual’s local and administrative experience. Each member presented and shared their answers with their group members and discussed the problem of participation rates. They also identified other problems and possible solutions in their discussion. Additionally, they mentioned several relevant points regarding survey conduction during the COVID-19 pandemic.

After the group discussion, they were asked to write about the topics addressed in a 2 × 2 matrix with the following quadrants: (1-1) problems that affect the participation rate, (1-2) measures that increase the participation rate, (2-1) can be controlled, and (2-2) cannot be controlled. The information of the matrix, including examples, is shown in Table 2.

Once the matrices were completed, each group was asked to analyze their matrix and list similar sentences under the same code. Codes with similar meanings were then combined into a single category, and each category was subsequently named. All members were involved in the categorization and agreed on the codes and categories. At the end of this process, the contents produced by the five groups were combined into a presentation titled, “Controllable Measures to Improve Participation Rates.”

### 2.3. Data Analysis

The data analysis was performed manually by the researchers using the Jiro Kawakita (KJ) method. The KJ method is a qualitative research strategy developed by Jiro Kawakita [27,28] to categorize data. The process involved three main steps:

- Step 1: Reading the material produced by the five groups several times to obtain an overall sense of the data. The researchers also carefully read the codes and categories for each group.
- Step 2: Grouping similar codes into categories and giving these groups a name based on the information given by the health personnel of local governments. Categories with a similar meaning can be given different names by each group. When this was the case, the researchers adopted the category name that was the easiest to understand. Finally, the codes and categories produced by the five groups were combined into one complete model, and the number of groups that used each code and category was written in parentheses after each item.
- Step 3: Performing a consensus analysis of the final results.

### 2.4. Statistical Analysis

The study subjects’ characteristics were summarized as mean and standard deviation (SD) or percentages, and their differences between area were analyzed using *t* test, analysis of variance, or χ^2^ test. All statistical analyses were carried out using SAS software, version 9.2 (SAS Institute, Inc., Cary, NC, USA). A probability (*p*) value of <0.05 was considered statistically significant.

## 3. Results

Table 3 shows the characteristics of the health personnel, including their government agency of origin, area, professional qualifications, and administrative experience.

Fifteen persons came from thirteen prefectures and six from cities with public health centers and special wards. Two persons were from Hokkaido and Tohoku, six from Kanto, six from Hokuriku and Tokai, three from Kinki, three from Chugoku and Shikoku, and one from Kyusyu and Okinawa. The mean age was 35.1 years. Sixteen persons were registered dietitians and four were public health nurses. Additionally, sixteen persons had 5–9 years of administrative experience, and five persons had more than 10 years. There were no significant differences in these characteristics between areas.

Table 4 shows the results of the workshop for all five groups, including the codes, categories, and examples of the measures suggested by the health personnel. The examples given in the table were actually implemented by local governments and coded by the health personnel. The group discussions revealed 12 categories of initiatives that could be implemented to improve the survey participation rate: 1. standardization of survey methods, 2. securing investigator skills, 3. survey organization, 4. venue setting, 5. accessing target households, 6. time of survey, 7. responses during the investigation, 8. confirming meal contents reported in the Nutritional Intake Status survey, 9. rewards/incentives, 10. possible rewards, and 11. feedback on survey results. The groups also created a 12th category titled “Survey practices during the COVID-19 pandemic.” The “Rewards/incentives” category was divided into two factors to address different issues: the first focused on the need to reward participants and how this should be carried out, while the second involved a discussion of the specific rewards that may be most appealing to participants.

## 4. Discussion

Japan’s NHNS is managed by the MHLW and is implemented by public health centers that have the resources to conduct it on a national level. The professionals who conduct the survey include dietitians who are responsible for taking dietary records and public health nurses who carry out health measurements [15,29].

The MHLW has developed a manual and protocol for the public health centers that conduct the survey to ensure the standardization of survey methods and data collection. However, the number of people who agree to participate in the survey is decreasing in recent years [16,17]. This study identifies methods for improving the participation rate based on the perspective and experiences of professionals in charge of the field survey. These methods could also be feasibly implemented by other local governments.

The present study revealed 12 categories of actions that could be implemented to improve the survey participation rate, including ideas that contemplated the restrictions associated with COVID-19. Since it has not been identified how much the actions affect the participation rate, it is necessary to conduct quantitative studies in the future. Studies conducted in other countries reported that these efforts may help increase the participation rates of sampled persons [21,22,23,30]. It is difficult to compare the results of this study with those of other countries; as few annual surveys are conducted by public health centers and local governments, such as the NHNS in Japan. Nevertheless, similar studies, such as a Finnish investigation of a migrant health survey, reported participation rate issues that resembled those observed in this investigation [30]. In addition to some solutions that were reported in other investigations, this study identified some promising measures to improve survey participation rates that have not been suggested in the literature.

During group activities in this study, there was much discussion about the investigators conducting on-site surveys. Ultimately, the health personnel agreed that more experienced investigators would be better equipped to deal with the different situations they may encounter during home visits. In other words, they may be the most suited to interact with target respondents. This suggestion is supported by a previous study, which found that the way interviews were conducted by investigators affected the quality of response data collected [31,32].

Additionally, participants agreed that experienced and inexperienced investigators should work in pairs to improve the skills of those with less experience. Furthermore, in the survey organization category, they pointed out that cooperation with organizations and groups in the community could improve participation and participation rates. Additional measures proposed were scheduling appointments at the convenience of the target participant and conducting the investigation in a flexible manner according to respondents’ needs.

Regarding the survey method, the health personnel pointed out that it was important for the target subjects to fully comprehend the survey before administration. In previous studies of home-based surveys, contacting survey participants by telephone [33] and mail [34] before the survey visit has proved successful in boosting participation. The National Health and Nutrition Examination Survey (NHANES) [35] in the United States is another example of a home-based survey. In this case, information on the survey is available on its website, where the target population is given orientations such as the following: “Interviewer will come to your home to talk to you about the survey. This interviewer should present an identification badge, which identifies this person as a health representative working on the NHANES” [35].

Japan’s MHLW currently provides general information about the NHNS on its website but does not provide a message directly to the people expected to respond to the survey. Each local government is tasked with explaining the NHNS to the local population. However, a message from the national government to those surveyed might help improve participation rates.

The NHNS is mainly conducted through home visits and face-to-face surveys at specific venues, but some local governments have begun to accept survey forms returned by mail to increase convenience for respondents. Previous studies reported that participation rates are higher when participants are given a physical paper questionnaire as opposed to a link to an online or web-based survey [7,22,36]. However, there are reports that the participation rates in each format may depend on the characteristics of respondents [37]. Furthermore, electronic surveys may be more convenient for participants with disabilities [38]; therefore, it may be effective to have the subjects select a response method from a range of different formats [22].

Regarding the Nutritional Intake Status survey, the health personnel agreed that it was devised to provide accurate information on the meal contents of respondents. In recent years, changes in the food environment have been significant, including an increase in food types, processed foods, and ready-made foods, and the diversification of products for local needs. The dietary content and meal forms available in Japan have also diversified. Therefore, it is important to ensure that the dietary survey in the NHNS can access information that is as accurate as possible. The results of this study revealed that some ways to increase this accuracy could be by requesting to see the packaging of food products and school lunch menus.

In the study, many issues related to rewards and incentives were also discussed. A Cochrane review has reported that rewards and incentives have a meaningful impact on survey cooperation rates. In several other reports, monetary incentives, such as gift certificates, were effective at increasing participation when many nonparticipants in the survey have a low income and are from younger generations [39,40]. Studies have also found that it can be effective to pay the reward before conducting the survey [41]. Both suggestions were mentioned by health personnel who participated in this study.

Our study had some potential limitations. First, only 21 individuals in 19 local governments among the 157 local governments were involved. There were also variations in the regional characteristics and years of administrative experience between the members in this investigation, even if there were no differences between areas. These differences may have influenced individual remarks and group dynamics. However, the results obtained in this study were similar to those observed in workshops conducted from 2018 to 2020, in which 40 local governments participated. The results of this study are unlikely to have been significantly affected by these limitations.

Another potential concern is that the qualitative methodology used prevents us from generalizing the results to other Japanese prefectures. Nevertheless, these findings have provided a basis for future quantitative investigations. A significant finding of this study was the interest of local government personnel in improving the quality of surveys. These findings indicate that the planning of this type of survey should consider community characteristics and, above all, the needs of investigators in charge of conducting the survey.

We believe that the measures suggested in this study constitute novel alternatives for improving survey participation rates and will be applicable to future revisions of the NHNS protocols. However, we have not examined the cost effectiveness of these ideas on the participation rate. This issue should be investigated in future studies.

## 5. Conclusions

This study aimed to identify the successful measures used by local government health personnel to improve the participation rate of the NHNS survey. The data were collected through a workshop for survey workers held at the training course of NIPH. The contents were coded and grouped to create categories. For data analysis, researchers combined and reviewed all codes and categories. The results yielded the following 12 types of actions: 1. standardization of survey methods, 2. investigator skills, 3. survey organization, 4. venue setting, 5. accessing target households, 6. time of survey, 7. responses during investigation, 8. confirming meal contents reported in the nutritional intake status survey, 9. rewards/incentives, 10. possible rewards, 11. feedback on survey results and 12. survey practices during the COVID-19 pandemic. These findings represent viable initiatives for local health personnel to increase participation rates and quality of responses for NHNS. A result of the data analysis from this workshop could provide high-quality insights with practical applications for improving participation rates, and the quantitative data might be collected based on the results in future.

## Figures and Tables

**Table 1:** Workshop procedure.

	Content	Time
0	Skill acquisition and group work exercises conducted using Zoom.	60 min
1	Based on the semistructured questionnaire that the health personnel answered before the training, each person filled out a worksheet with their opinions on the following two topics: (1) problems that seem to affect the participation rate of the National Health and Nutrition Survey and (2) measures that could be used to improve the participation rate.	20 min
2	Members carried out an online group exercise based on the two abovementioned topics. Five groups were set up for this purpose. Each member presented and shared their answers with their group and discussed the problem of participation rates. They also identified other problems and possible measures in their discussion. Additionally, they mentioned several relevant points regarding survey conduction during the COVID-19 pandemic.	20 min
3	Members were asked to write about the topics addressed in a 2 × 2 matrix with the following quadrants: (1-1) problems that affect the participation rate, (1-2) measures that increase the participation rate, (2-1) can be controlled, and (2-2) cannot be controlled.	30 min
4	Once the matrices were completed, each group analyzed their matrix and listed similar sentences under the same code. Codes with similar meanings were combined into a single category, and each category was subsequently named. All members were involved in the categorization and agreed on the codes and categories.	20 min
5	The contents produced by the five groups were combined into a presentation titled, “Controllable Measures to Improve Participation Rates.”	50 min

**Table 2:** Matrix of the topics addressed.

	Can Be Controlled ^†^	Cannot Be Controlled ^†^
Problems that affect the participation rate	Respondents are not rewarded for their participation ^‡^	No community leader in the area can persuade cooperation with the survey ^‡^
Factors that increase the participation rate	Training investigators in interviewing techniques to help them obtain accurate answers from respondents ^‡^	There are many apartments in the survey area ^‡^

^†^ The problem/measure can/cannot be controlled by the governmental agency. ^‡^ Example sentences.

**Table 3:** Characteristics of the health personnel including their government agency of origin, area, professional qualifications, and administrative experience (n = 21).

			No. ^‡^
Government agency	Classification	Prefecture	15
		Cities with public health centers and special wards	6
	Area	Hokkaido and Tohoku	2
		Kanto	6
		Hokuriku and Tokai	6
		Kinki	3
		Cyugoku and Shikoku	3
		Kyusyu and Okinawa	1
Personnel	Age (years) ^†^	Mean	SD
		35.1	6.5
	Professional qualifications	Registered dietitian	16
		Public health nurse	4
		Others	1
	Administrative experience (years)	5–9	16
		≥10	5

^†^ SD; standard deviation. ^‡^ number of persons.

**Table 4:** Controllable measures to improve participation rates of the National Health and Nutrition Survey.

Category	Code (Number of Groups that Cited the Code)	Examples of Measures that Have Been Implemented to Improve Participation Rate
1. Standardization of survey methods	-Create materials that are easy to use and applicable to the situation of local governments (2)	-Creating individual materials to return the results to each subject after the survey
	-Implement the same survey methods across public health centers (1)	-Unify survey tools among health centers
2. Securing investigator skills	-Improving the skills of inexperienced investigators by pairing experienced and inexperienced investigators (5)	-Encouraging experienced investigators to advise inexperienced ones and point out issues to be resolved led toward improvements in performance
	-Clarifying the work conducted by the investigator (2)	-Explaining the specific work contents to the investigator
	-Understanding the role of investigators (1)	
3. Survey organization	-Securing the cooperation of the residents’ association chairman ^†^ (5)	-Learning about the local situation from the chairman of the residents’ committee and using it to adjust the survey venue and schedule
	-Securing the cooperation of the municipality (4)	-National surveys seem like an unfamiliar procedure, but showing that the municipality is involved in the process may encourage residents to participate and help them understand the procedures involved in the survey
	-Securing the cooperation of the ward mayor (3)	-Requesting cooperation from the ward mayor
	-Ensuring cooperation among public health centers (2)	-Promoting cooperation between health centers to train young personnel in charge of the survey
	-Ensuring the cooperation of the Dietetic Association (1)	-It was easier to carry out the survey if there was cooperation and information sharing with the Dietetic Association network
4. Venue setting	-Securing an easily accessible place (2)	-Renting a local community center
5. Accessing target households	-Visiting the target households (5)	-Explanations regarding the survey were given in door-to-door visits
	-Explain the benefits of the survey (5)	-Informing the subjects in advance that they would receive the survey results
	-Talk to respondents in an easy-to-understand way (4)	-Giving respondents a leaflet that clearly described what the survey results would be used for
	-Visiting the target household more than once (3)	-Visited each household multiple times to explain the survey and conduct the dietary survey
	-Informing residents in advance (3)	-Providing advance notice of the survey by mail
	-Devising a way of explaining the survey (2)	-Informing citizens about the NHNS for their cooperation
6. Time of survey	-Administering the survey in the evening if that is convenient for participants (4)	- Survey visits were also conducted in the evening
	-Scheduling survey interviews based on the respondents’ availability (3)	-Scheduled survey visits at the convenience of participants
	-Securing investigators for longer periods of time (2)	-Recruiting investigators who could work in the evening
	-Selecting the questionnaire format according to participants’ convenience (2)	-Collecting survey forms in the way that was most convenient to participants, such as in-person visits or through the mail
7. Responses during the investigation	-Providing flexible responses to the needs of the target person (1)	-Responding flexibly, such as by listening to the needs of the target person
8.Confirming meal contents reported in the Nutritional Intake Status survey	-Showing the package of the products they have eaten (3)	-Showing the packaging of frozen foods and sweets
	-Giving participants additional notepaper (2)	-Asking participants to write down notes or questions about dietary records to facilitate their communication with investigators during the survey visits
	-Taking a picture of the meal eaten (1)	-Asked participants to take pictures of frozen foods and sweets they had recently consumed
	-Not asking the target person if the investigators can confirm their answers (1)	
9. Rewards/Incentives	-Giving gifts to respondents who complete all surveys (3)	-A small but appealing reward would be given to those who completed the entire survey
	-Giving (part of) the reward before the investigation (2)	-Give the reward early; it was easier to secure participation by sending participants a reward before returning the results of the survey
10. Possible rewards	-Local gift certificate/product (2)	
	-Measuring spoon/scale (2)	
	-Large products (1)	
	-Book (1)	
	-Changing the contents of the reward every year (1)	
11. Feedback on survey results	-Providing comments and leaflets regarding the results (4)	
12. Survey practices during the COVID-19 pandemic	-Responding online to the Lifestyle Status Questionnaire to reduce face-to-face contact (2)	
	-Reducing face-to-face contact by distributing videos to explain the survey (2)	
	-Conducting surveys at different times to avoid crowding (2)	
	-Since it is difficult to hold group meetings to request participation in the survey, this information should be given in door-to-door visits (1)	
	-Increase the use of mail surveys (1)	
	-Find a large venue (1)	
	-Set up a reservation system for conducting the Physical Status Questionnaire (1)	

The numbers in brackets represent the number of the groups making that recommendation. ^†^ The residents’ association chairman means the representative or responsible person of the residents’ association in the area, and organizes local events such as summer festivals, new year events and disaster drills.

## Data Availability

The data that support the findings of this study are available from NIPH, but restrictions apply to the availability of these data and so are not publicly available. Deidentified and aggregated data are however available from the authors upon reasonable request and with permission of NIPH.

## Supplementary Materials

The following supporting information can be downloaded at: https://www.mdpi.com/article/10.3390/nu14193906/s1, Figure S1: Survey system of National Health and Nutrition Survey.
