# Supplementary figures and images for: Possible Measures to Improve Both Participation and Response Quality in Japan’s National Health and Nutrition Survey: Results from a Workshop by Local Government Personnel in Charge of the Survey
Source: Nutrients. 2022 Sep 21;14(19):3906. doi: 10.3390/nu14193906 (PMC9571403; doi:10.3390/nu14193906)

Figure S1 Survey system of National Health and Nutrition Survey

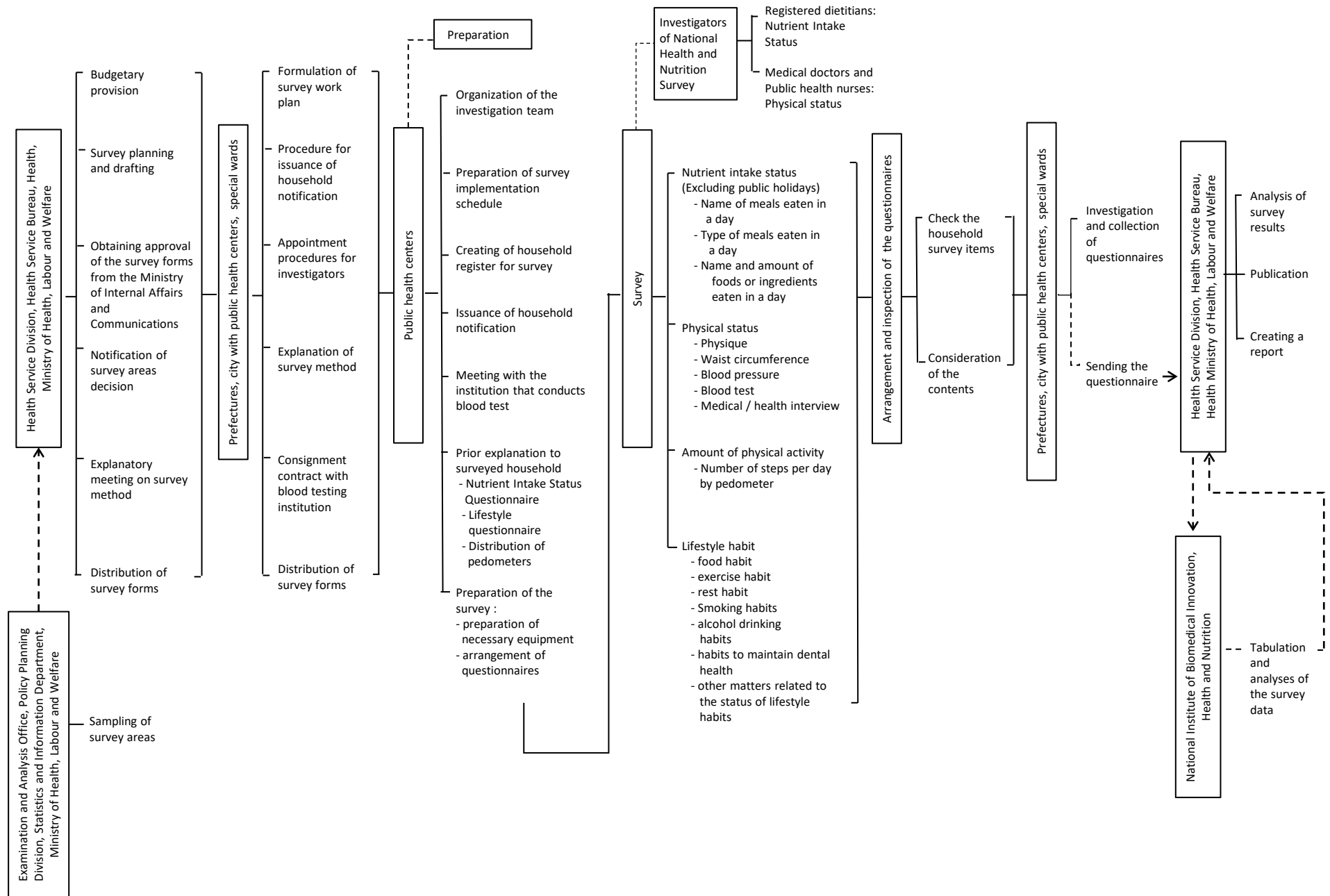

Supplement: Supplementary file 1 [file nutrients-14-03906-s001.zip › nutrients-1926670-supplementary.pdf]
